# School-based screening for high blood pressure in children and adolescents: a systematic scoping review

**DOI:** 10.1038/s41440-025-02542-z

**Published:** 2026-01-21

**Authors:** Jonathan P. Glenning, Freya Sheeran, Catherine Quinlan, Jonathan P. Mynard

**Affiliations:** 1 Heart Research, Murdoch Children’s Research Institute, Parkville, VIC Australia; 2 Department of Paediatrics, University of Melbourne, Parkville, VIC Australia; 3 Department of Cardiology, Royal Children’s Hospital, Parkville, VIC Australia; 4 Department of Nephrology, Royal Children’s Hospital, Parkville, VIC Australia; 5 Department of Nephrology, Children’s Health Ireland, Dublin, Ireland; 6 Department of Biomedical Engineering, University of Melbourne, Parkville, VIC Australia

**Keywords:** Hypertension, Preventive medicine, Screening, Blood pressure, Implementation

## Abstract

School-based programs represent a potential avenue for conducting population-wide paediatric blood pressure (BP) screening. The aim of this review was to systematically scope peer-reviewed literature reporting school-based BP screening, with respect to measurement protocols, diagnostic process coverage, and implementation considerations. Only peer-reviewed articles in English across PubMed, OVID Medline and OVID Embase were included. Two authors independently screened the article titles and abstracts prior to undertaking a full-text review. All disagreements were resolved through discussion and agreement. From each study, four categories of information were extracted: general information, BP measurement methodology, diagnostic process coverage, and implementation strategies. Each article was then assigned to one of three categories regarding the stated or implied study objectives: general school-based research incorporating BP measurement, hypertension prevalence studies, or hypertension screening studies. Of the 112 articles meeting the inclusion criteria, only 17 were categorised as hypertension screening studies. Within these, there was substantial variability in BP measurement techniques and adherence to the diagnostic process recommended by the American Academy of Pediatrics. Additionally, there was minimal reporting on implementation strategies. A pragmatic, standardised protocol for school-based BP screening is needed that includes recommended measurement methods, considers the trade-offs (in terms of feasibility and economics) of covering more or less of the diagnostic process in schools vs health care settings, and covers approaches to optimise implementability.

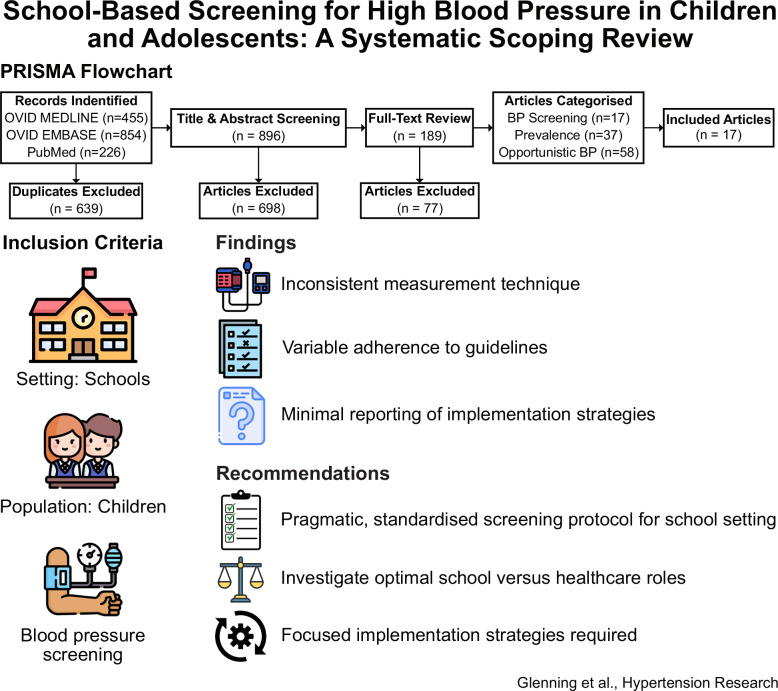

## Introduction

Children and adolescents with high blood pressure (BP) have an increased risk of fatal and non-fatal cardiovascular events by mid-adulthood, with intermediate markers of cardiovascular disease initiation already emerging in youth [1–4]. Importantly, addressing high BP through clinical management has been shown to reduce the intermediate markers of cardiovascular risk [5–8]. Consequently, regular BP screening is recommended in all paediatric clinical guidelines [9, 10], with the rationale that earlier identification of high BP facilitates earlier intervention and long-term risk reduction [11, 12].

In practice, adherence to paediatric screening guidelines is relatively low [13, 14], with studies from the United States of America identifying barriers such as: (1) limited understanding and prioritisation of high BP in children [15]; (2) the perception that measuring BP in children is anxiety-inducing with poor tolerability [16]; or (3) the perception of practitioners that it is difficult to measure BP in children, with adherence to best-practice measurement methods challenging in real-world settings [16–18].

School-based programs represent a potential avenue for conducting population-wide paediatric BP screening. Whilst the 2017 American Academy of Pediatrics (AAP) guideline [9] noted that there is some evidence that school measurements can be reliable [19] and that longitudinal follow-up is feasible [20, 21], school-based diagnosis was not recommended due to insufficient evidence and lack of established protocols (at the date of publication). Nevertheless, school-based BP measurement was seen to be ‘a useful tool to identify children who require formal evaluation’ [9].

These comments highlighted the need for further evidence to be generated and the development of a consistent and reliable protocol for school-based BP screening. However, three key questions arise when considering such a protocol:

First, how much of the diagnostic process should be covered in schools? It is conceivable that a school-based program could include an initial BP measurement followed by referral of children with high readings for follow-up. Or, a program could include more of the diagnostic process, such as multiple measurements with/without multiple encounters, and/or 24-h Ambulatory BP Monitoring (ABPM) to rule out white coat hypertension. However, the merits of greater or lesser coverage of the diagnostic process in a school setting have received little attention – for example, issues of feasibility, appropriateness, and burden on schools and health care settings should be considered.

Second, what specific BP measurement protocol will maximise accuracy and acceptability, commensurate with the chosen coverage of the diagnostic process? For example, if the aim is to quickly identify children and adolescents with possible high BP before referring them on to be properly assessed, then would a single automated BP measurement with a shortened one to 2-min rest period suffice? Or, if a more thorough assessment were to be performed in the school environment, then a minimum of three manual BP measurements with the full guideline specified 5-min rest period may be more suitable.

Third, what implementation considerations are likely to impact the design of a school-based BP screening protocol? Careful planning and embedding of implementation strategies are likely to be critical to achieving acceptability, scalability and sustainability.

Therefore, the overarching aim of this scoping review is to provide an updated review of studies reporting school-based BP screening and to use this as a platform to explore the key questions of measurement protocols, diagnostic process coverage, and implementation considerations. Specific aims were to: (1) determine the extent and nature of published scientific literature on studies which identified children and/or adolescents’ hypertension status through BP measurement in a school setting; (2) identify the specific BP measurement methodologies used and the coverage of the diagnostic process as described in the AAP guideline; and (3) identify and synthesise the implementation strategies that were employed to facilitate BP screening in a school setting.

## Methods

This systematic scoping review was conducted in accordance with the scoping review guidance published by Levac et al[22]. and was reported in accordance with the PRISMA Extension for Scoping Reviews Reporting Checklist (see Supplementary Materials Section 1).

### Search strategy

Three databases were searched: OVID Medline, OVID Embase, and PubMed. The search criteria used for each database are provided in the Supplementary Materials (Sections 2–4). Briefly, inclusion criteria were as follows: (1) systemic arterial BP was measured in children/adolescents, (2) BP measurement occurred in the school setting, (3) BP measurement methodology, such as measurement method or patient preparation, was described, and (4) hypertensive status was classified and reported. The search was limited to peer-reviewed articles published in English between 01 January 2012 to 19 December 2024 (the search date). Conference abstracts, case reports, comments, editorials and letters were excluded.

### Study selection criteria and screening process

One author (JG) completed the search of each database and deposited all relevant articles found into Covidence (Veritas Health Innovation, Melbourne, Australia). Two authors (JG and JM) independently screened the article titles and abstracts after duplicate records were removed. In order to determine eligibility for inclusion, a full-text review was then undertaken by two independent researchers from a group of three authors (JG, JM and FS). All disagreements were resolved through discussion and agreement between the two reviewers.

### Extraction/analysis plan

Three categories of data were extracted from the included articles by a group of three of the authors (JG, JM and FS):

1. General information: study design, population, number of participants, and type of school(s).
2. BP measurement methodology and screening protocol: how was BP measured, how many times, where was BP measured, details of any initial rest period, interval between measurements, what guidelines were used to define BP status, if and how screening outcomes were communicated to parents and/or healthcare practitioners, and other relevant details related to the methods used for BP measurement.
3. Implementation strategies: strategies used to make BP measurement easier or to improve the efficiency/effectiveness of the program.

Each article was then assigned to one of three categories: (a) general research studies conducted opportunistically in a school population where the main focus was not screening for high BP; (b) studies assessing the population prevalence of hypertension in a school setting (reporting population statistics only, with no stated or implied intention of clinical referral or follow-up of individuals with identified hypertension); or (c) studies where BP was measured in a school setting with the intent of identifying individuals with hypertension (i.e. screening with the intention of clinical diagnosis, referral or follow-up). For the purposes of this review and its focus on screening, only the articles that fell into the third category were subjected to detailed analysis and discussion.

Data were exported and cleaned in Microsoft Excel (Microsoft, Redmond, USA) and synthesised using a mixed methods approach, with NVivo (Version 14, Lumivero, Denver, USA) for qualitative data, and R (v4.2.1; R Core Team) & RStudio (v2023.03.1 + 446; Posit Team) for quantitative data. Counts and frequencies were calculated to describe quantitative variables, such as what BP measurement technique was used or the country where the study was undertaken. Qualitative data were coded, and a content analysis was undertaken in NVivo.

## Results

**Fig. 1:**
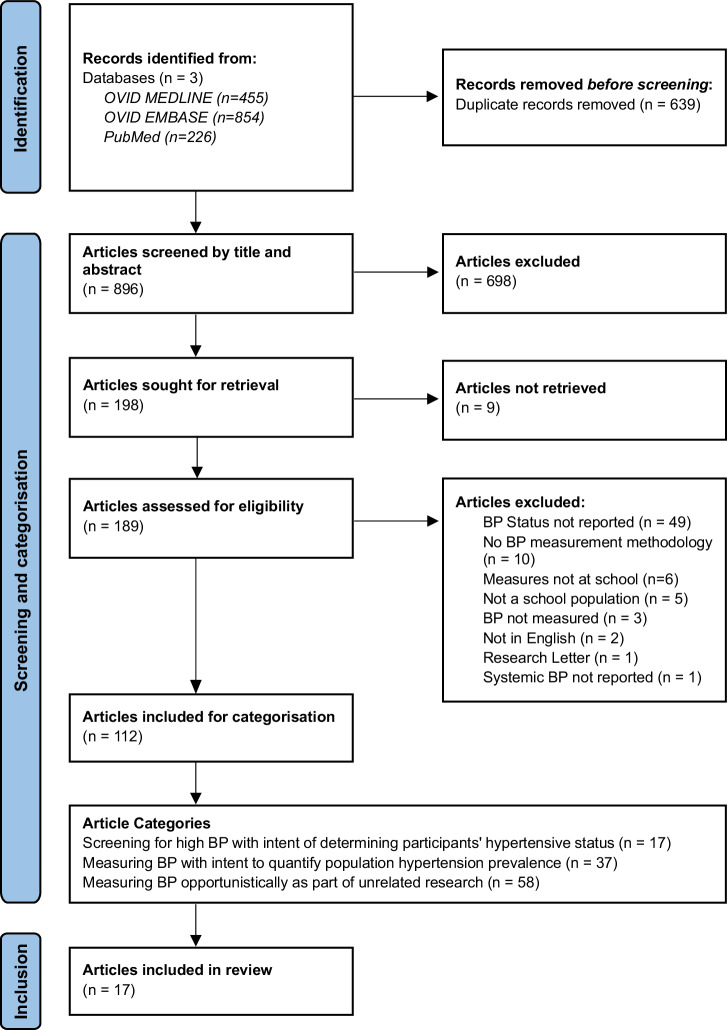
PRISMA flowchart of identified, screened, included and categorised articles for review

Searching the three databases resulted in 896 articles (after 639 duplicates were removed). After title and abstract review, 698 articles were excluded, and after full-text retrieval and review, a further 77 articles were excluded. Further details are provided in the PRISMA diagram in Fig. 1.

Of the 112 articles included after full-text review, 58 were judged to be opportunistic school-based research incorporating BP measurement but unrelated to hypertension screening, 37 intended to quantify hypertension prevalence with a population focus, and 17 articles involved screening for high BP with the intent of identifying individuals with hypertensive BP for clinical diagnosis, referral or follow-up. The latter were included in this scoping review.

The programs described in these 17 articles took place across 11 countries, with three in WHO-defined lower middle-income countries [23–25], four in upper middle-income countries [26–29] and the remaining 10 in high-income countries [30–39]. A majority (13, 76%) of the articles were completed specifically as part of research studies [23–31, 35–37, 39], whilst four (24%) were reporting on established programs (e.g. embedded government-funded school health programs) [32–34, 38]. Additionally, two articles (12%) were conducted in children alone (<13 years) [27, 28], three (18%) were conducted in adolescents alone (≥13 years) [24, 34, 35] and 12 (71%) in both [23, 25, 26, 29–33, 36–39]. The majority (14, 82%) were conducted either partially or solely in secondary schools (ages 14–18 years) [23–26, 29–36, 38, 39], with five (29%) being conducted either partially or solely in middle schools (ages 12–14 years) [29, 30, 32, 37, 38] and six (35%) either partially or solely in primary schools (ages 5–12 years) [26–28, 36, 38, 39].

### Blood pressure measurement methodologies

When considering the 17 articles with screening intent, BP measurement in children and adolescents was conducted using an automated device in 14 (82%) [23–26, 28–35, 37, 38], and the manual auscultatory method in five (29%) [25, 27, 36, 37, 39]. Two articles used automated BP measurement initially before confirming high readings with manual BP measurement [25, 37].

#### Rest period prior to measurement

Nine (53%) articles specified that BP was measured after a rest period, with six (67%) using a period of 5 min [23, 26, 29, 35, 36, 39], one (11%) article using 3 min [30], one (11%) using 10 min [31] and one (11%) using 15 min [24]. The remaining eight (47%) articles did not report whether there was a rest period.

#### Number of BPs per encounter

There was substantial variability in the number of times BP was measured per encounter across the various articles, including three times in eight (47%) articles [23, 25, 26, 29, 31, 35, 36, 39], two to three times in three (18%) [28, 32, 33], twice in two (12%) [27, 30], one to two times in two (12%) [24, 37], one to eight times in one (6%) [34], and only once in one (6%) article [38].

#### Number of BP measurement encounters

There was also variability in the number of BP measurement encounters across the articles, with two (12%) articles measuring BP across three encounters [32, 33], two (12%) across two encounters [23, 36], three (18%) across one to three encounters [25, 26, 29], one (6%) across one to two [28], and nine (53%) articles having only one encounter [24, 27, 30, 31, 34, 35, 37–39]. Of the eight (47%) articles that measured BP across more than one encounter, six (35%) only completed a subsequent encounter for those who had an elevated BP on the first encounter [23, 25, 26, 28, 32, 33]. In the article by Liu et al. [29] there were at least two encounters (across two of the six participant centres tested; in the other four centres, all participants only underwent one encounter), and only those with elevated BPs on the second encounter underwent a third encounter. In the article by Kollios et al. [36], all participants had one encounter, with a randomly selected subset undergoing a second encounter.

#### Rest period between measurements

11 (65%) articles specified that there was a rest period between BP measurements in a single encounter, with five (45%) using a period of one minute [23, 28, 30, 32, 33], three (27%) using a period of 5 min [27, 29, 31], two (18%) using a period of 2 min [25, 36], and one (9%) using a period of 10 min [24]. The remaining six (35%) articles did not specify a rest period between measurements.

#### Cuff and child positioning

Nine (53%) articles specified that BP was measured on the right arm [23, 25, 26, 31–34, 36, 39], one (6%) on the left [30], and three (18%) both (one where BP was initially measured in both arms and the higher used, and two where the participant’s preferences were sought and followed) [24, 29, 35]. The remaining four (24%) articles did not specify which arm was used. Eleven (65%) specified that BP was measured in the seated position [23, 24, 26, 29–33, 35, 36, 39], with five (29%) of those stating that this was with the participants’ back and arms supported, and legs uncrossed with feet touching the ground [24, 26, 29, 30, 36]. These details were not provided in the remaining six (35%) articles. Furthermore, in three articles (18%), the participants were given further instructions such as to empty their bladder prior to measurement, to be relaxed and keep quiet/not talk, not use mobile phones, or not to smoke or have any stimulants during the previous hour prior to measurement [24, 26, 30]. The remaining articles did not provide any details on additional preparatory instructions.

#### Ambulatory blood pressure monitoring

24-h ABPM was undertaken in two (12%) of the articles and completed on a normal school day [23, 36]. In both articles, the non-dominant arm was used, and participants were instructed to act normally. In the article by Nsanya et al. [23], participants were instructed to maintain a diary with sleep/wake times and any periods of activity or distress noted in the diary were accounted for when interpreting the ABPM results. Kollios et al. [36] directed participants to rest or sleep between midnight and 6 am (even if they were usually out of bed earlier than this) and to maintain their usual activities (though avoiding any strenuous activity or daytime sleeping) between 8 am and 10 pm. An adequate 24-h ABPM was defined as at least 20 readings during the day and seven at night [23], or at least 72 valid BP measurements [36].

### Diagnostic process coverage

The BP measurement and classification guidelines used were specified in 16 of the 17 (94%) articles, with eight (50%) using the AAP 2017 guidelines [23, 26, 27, 29, 30, 32, 37, 39], seven (44%) using the Fourth Report 2004 guidelines [25, 26, 28, 32–34, 38], four (25%) using the European Society for Hypertension 2016 guidelines [24, 35, 36, 39], and one (6%) using the BP reference standards for Chinese children and adolescents [26]. Three (19%) of these articles used more than one guideline for the purpose of comparing them [26, 32, 39].

**Fig. 2:**
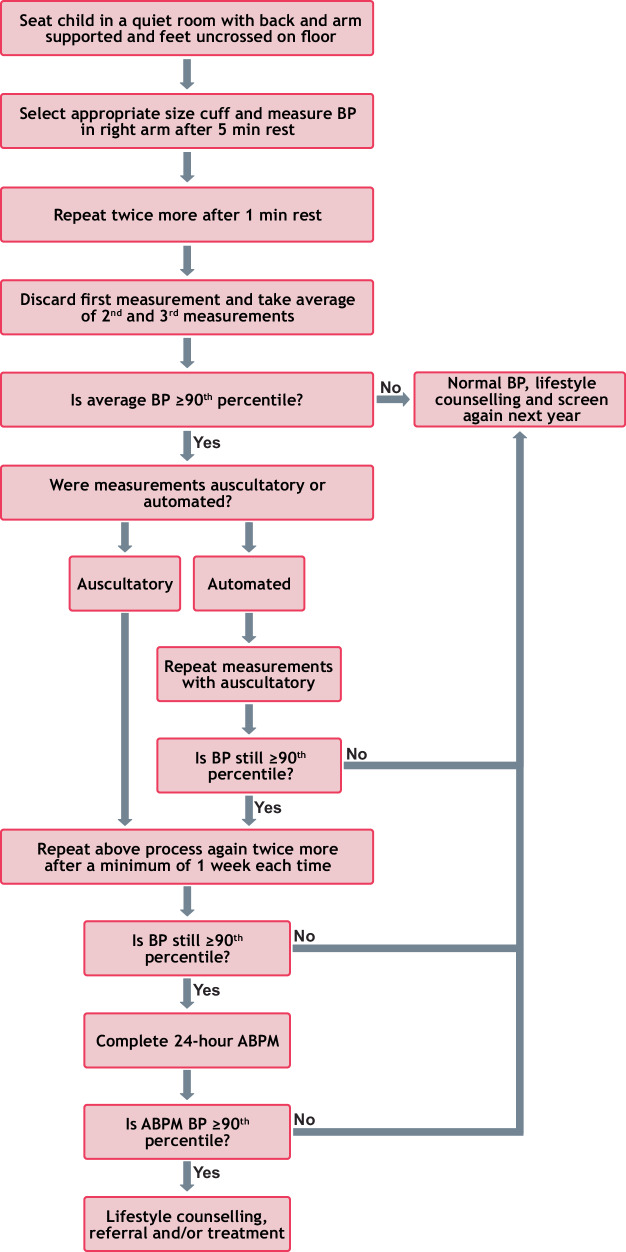
Flowchart of process for the diagnosis of hypertension in children and adolescents as per the American Academy of Pediatrics clinical practice guideline 2017

**Fig. 3:**
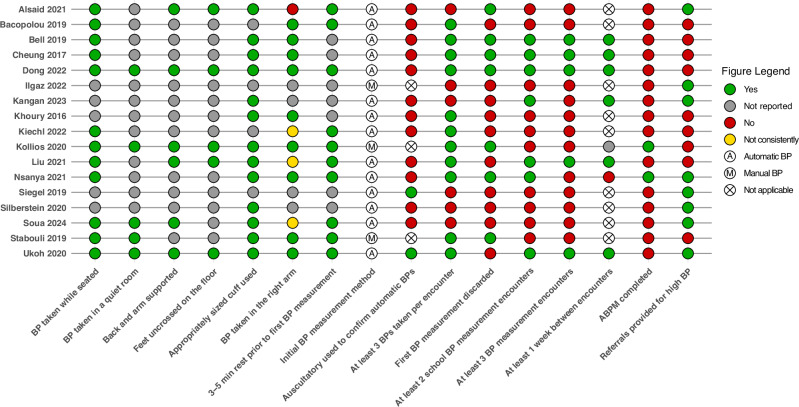
Summary of individual article methodology and coverage of the American Academy of Pediatrics diagnostic process. Figure visually denotes each reviewed article’s blood pressure measurement methodology against the best practice guidance laid out in the American Academy of Pediatrics clinical practice guideline 2017, as well as each article’s coverage of each step of the diagnostic process laid out in the same guideline

Across the 17 articles, three (18%) covered almost the entire diagnostic process, as per the 2017 AAP guideline (Fig. 2), though each had some missing elements (recognising that one of the 17 articles was published prior to 2017). Kollios et al. [36] only measured BP across two encounters (one at school as an initial screen and then one subsequently offsite in a medical setting), and did not report if there was a referral for further management of high BP. Dong et al. [26] only used automated BP, did not perform ABPM and also did not report if referral for high BP management occurred. Ukoh et al. [25] did not discard the first BP measured in each encounter, and did not perform ABPM. The remaining articles had variable coverage of the diagnostic process, with most either not meeting or omitting details about key parts. As an example, only eight articles (47%) explicitly reported or implied that referral was made to health professionals for follow-up [23–25, 27, 28, 30, 37, 38], with most providing minimal detail about how this was conducted. The remainder did not report this outcome [26, 29, 31–36, 39]. The specific details of each article’s coverage of the diagnostic process outlined in the AAP guideline are presented in Fig. 3.

### Implementation strategies

Various implementation strategies were employed. The three main categories were stakeholder engagement (engaging with parents/schools through letters and discussions), education (of students and/or parents), and strategies for easier BP measurement/anxiety-reduction (familiarisation with BP measurement, casual clothes for assessors).

#### Stakeholder engagement

Of the 17 articles, all engaged with parents of prospective participants in some way, such as letters (along with consent or opt-out forms) or information sessions. Schools also typically provided consent to participate in these studies, often after correspondence or discussions with the respective research teams.

#### Education

In one of the articles, education programs, with the goal of promoting healthy behaviours and habits to limit future hypertension, were delivered ahead of the measurements being taken [30]. In another article, education programs about healthy diets and physical activity were implemented in collaboration with parents, students and school staff [27].

#### Strategies for easier BP measurement

Strategies to make BP measurement easier were also undertaken in some articles. Two detailed that prior to commencing any measurements, each participant was familiarised with the BP measurement process [23, 39]. Nsanya et al. [23] also allowed the participant to start the automated device themselves, rather than the researcher. Another strategy reported by Dong et al. [26] was that staff undertaking the measurements were all dressed in casual clothes rather than more professional attire. Finally, Ukoh et al. [25] reported the use of an online calculator by the researchers to determine BP percentiles rather than the use of look-up tables.

## Discussion

This scoping review identified 17 articles from 11 countries with the objective of BP screening in schools. A key finding was the significant variability in BP measurement methodologies and adherence to clinical practice guidelines. While the majority of articles (82%) employed automated devices, there was considerable inconsistency in rest periods before measurements and the number of readings per encounter, with inconsistent reporting of the positioning of participants during BP assessment. Three articles performed close to the entire diagnostic process as recommended by the AAP [25, 26, 36], however the remainder had partial or inconsistent coverage. Implementation strategies included stakeholder engagement and educational interventions, along with methods to reduce anxiety during measurement. Given the small number of articles identified and their substantial variability in both method and reporting, there remains limited evidence regarding (1) how much of the diagnostic process is suitable to conduct in schools, (2) what is the most accurate and acceptable BP measurement protocol, and (3) what implementation approaches maximise effectiveness. However, in consolidating the existing evidence, this review highlights important considerations that warrant further investigation and evaluation for the implementation of school-based BP screening programs.

Important trade-offs must be considered when determining the most appropriate coverage of the diagnostic process within schools. Available guidelines were designed for a healthcare environment and completing the entire diagnostic process within the school environment could be overly resource-intensive and burdensome on school staff/resources, which are principally concerned with education rather than healthcare. Conversely, many articles in the literature highlight the importance of multiple measurements and multiple encounters to reduce false positives [20, 23, 40, 41]. Accordingly, a screening protocol with a single automated measurement prior to referral might be less resource-intensive and less burdensome on schools, but is likely to impose unnecessary burden on individuals who are misclassified and on the healthcare staff/system that investigates these false positive cases.

The question of the appropriate level of coverage of the diagnostic process, in conjunction with the variability in the protocols used in the reviewed articles, indicates a clear and present need for the development of a standardised protocol for BP screening in schools. It will be critical for any future protocol to be established in a pragmatic middle-ground between effectiveness and accuracy (the extent to which the program achieves its goals and how well the program correctly identifies students who do/do not have a high BP respectively), and acceptability and feasibility (for children and adolescents, their families and schools, for the healthcare system and for society more generally).

According to all paediatric guidelines, the auscultatory method is required to confirm a high reading with an automated device, as these devices have been shown to consistently overestimate BP in children and adolescents [9, 10, 42]. In this review, only 29% of articles used the auscultatory method either entirely, or to confirm measurements from an automated device. A drawback of the auscultatory method is that it requires substantial observer training and can involve inaccuracies related to inter-observer variability, cuff deflation rate and terminal digit preferences [43]. However, if the intention is for the school screening to only cover the initial assessment, as in Kollios et al. [36], then the use of an automated device is acceptable according to current guidelines [9]. Conversely, for a protocol designed for a more comprehensive screening process, confirmation with auscultatory measurements by trained operators is recommended.

Regarding BP measurement process, most of the articles reviewed did not fully adhere to (or report details on) guideline recommendations concerning participant positioning (seated, back and arm supported at heart level), rest periods (adequate rest while seated quietly, unmoving, prior to the first measurement as well as a reasonable gap between each measurement), and room conditions (quiet, relaxed, not filled with stressors) [9, 10]. Only three of the articles reported that they had adhered to all of these recommendations [25, 26, 36]. Non-adherence to these recommendations has a substantial negative impact on the accuracy of BP measurement [43–46]. Given the importance of accurate BP measurement regardless of whether the intent is solely initial screening or a more thorough diagnostic process, these recommendations should be reinforced to maximise the effectiveness of any future school screening protocol.

Another key aspect for future school BP screening protocols that requires further consideration is the role of 24-h ABPM. While ABPM was used in two of the reviewed articles, the facilitators and barriers to implementing ABPM in school settings have not been extensively investigated. Evidence in clinical settings suggests that children and adolescents regularly find ABPM to be intrusive, uncomfortable and sometimes painful [16], with Hamdani et al. [47] reporting that 32% of adolescent participants did not tolerate ABPM. Though these findings indicate substantial barriers are likely to exist in implementing ABPM in a school-based screening program, this must be balanced with the potential benefits to efficiency and program cost-effectiveness. For example, Swartz et al. [48] found that performing ABPM in all patients prior to referral for more detailed evaluation would yield a cost saving of approximately US$2.4 million per 1000 patients. Given these potential barriers and benefits, further exploration of tolerability concerns and potential facilitators is required prior to including 24-h ABPM within any standardised school-screening protocol.

When considering the findings of this review regarding pertinent implementation strategies for a school-based BP screening program, there was little reporting of and no evaluation of specific strategies. Each of the included articles reported varying (though mostly minimal) levels of stakeholder engagement despite its potential importance. The importance of parent and clinician stakeholder input was recently highlighted by both Zaidi et al. [49] and Baker-Smith [50], and while these were not specifically about school environments, it stands to reason that this would be pertinent in the school environment as well. Consequently, active communication with students, parents, and school staff is vital to the success of any program as it fosters trust, encourages higher participation rates, and consequently, promotes greater engagement with the program at large.

To maximise the benefit from and acceptability of a school-based BP screening program, a holistic approach beyond just the BP screening itself may be desirable. For example, Alsaid et al. [30] delivered educational sessions immediately prior to the BP measurements, reinforcing the importance of healthy behaviours for the children and aligning with the education goals of schools, a strategy identified previously in the literature as beneficial to the success of such a program [51, 52]. Another strategy was to consider methods for reducing anxiety related to the measurement process, such as familiarising students with BP equipment ahead of time or wearing casual clothing rather than formal clothing, as was reported in two of the reviewed articles [26, 39]. Creating more comfortable environments for the students is likely to enhance the reliability of the screening results by reducing white coat hypertension [43, 53], as well as facilitate acceptance of and engagement in the program by the students, parents, and staff. Other implementation strategies in other school-based health programs, such as clear data and referral pathways [54] and effective funding structures [55], are likely to be important considerations for implementation. However, given the paucity of research considering any type of implementation of school-based BP screening, further research is required to (1) identify the barriers and facilitators that will impact the effective, acceptable and sustainable implementation of such a program, and (2) develop implementation strategies to address those identified factors.

In regard to staffing, similar to childhood vaccination programs, for some countries, a school nurse-led program may be the most effective/efficient; however, for others without existing robust school nurse programs, it may be more preferable to have an external group that regularly visits schools [56]. A potential advantage of an external group could be greater staff experience/expertise in BP screening and protocols, whereas the advantages of school nurses conducting screening are that they are embedded in the school, well-known to students (which may reduce anxiety) and may be better placed to facilitate follow-up measurements and coordination of care for those identified at risk.

In light of the findings from this review, as well as these considerations regarding the diagnostic process and the optimal BP screening protocol, a pragmatic middle ground may be to include multiple measurements over two encounters in the school environment, with the third encounter and 24-h ABPM conducted in a healthcare setting as part of patient-focused diagnostic workup. The school portion of this could potentially be completed by school nurses (or those otherwise familiar with the students) after education sessions with both parents and students, with clear referral and data transfer pathways implemented to minimise the burden of follow-up. However, this is only one of many possible protocols and determining the optimal approach to both diagnostic process coverage and BP measurement protocol requires further research, as well as likely consideration of regional factors.

Limitations of this review are that only articles published in English across the three databases of PubMed, OVID Medline and OVID Embase were included. Additionally, due to incomplete reporting in the included articles, methodological steps in the articles may have been missed in cases where they were omitted or not clearly stated.

## Conclusion

School-based BP screening programs may be an effective method for screening children and adolescents for high BP. This review highlighted the substantial variability in BP measurement techniques and coverage of the diagnostic process, along with relatively superficial reporting of implementation considerations. There is a clear and present need for a pragmatic, standardised protocol for school-based BP screening to be described and evaluated, with particular consideration given to the scope of the school-based screening program and how it interacts with the healthcare system.

## Supplementary information

Supplementary information
